# Multiclass Determination of Endocrine-Disrupting Chemicals in Meconium: First Evidence of Perfluoroalkyl Substances in This Biological Compartment

**DOI:** 10.3390/toxics12010075

**Published:** 2024-01-15

**Authors:** Aritz Domínguez-Liste, Teresa de Haro-Romero, Raquel Quesada-Jiménez, Ainhoa Pérez-Cantero, Francisco Manuel Peinado, Óscar Ballesteros, Fernando Vela-Soria

**Affiliations:** 1 Analytical Chemistry and Life Sciences Research Group, Department of Analytical Chemistry, University of Granada, E-18071 Granada, Spain; adliste@correo.ugr.es (A.D.-L.); oballest@ugr.es (Ó.B.); 2 Instituto de Investigación Biosanitaria (ibs.GRANADA), E-18016 Granada, Spain; mariat.haro.sspa@juntadeandalucia.es (T.d.H.-R.); raqueluna77@gmail.com (R.Q.-J.); fervs@ugr.es (F.V.-S.); 3 Clinical Laboratory Management Unit, Hospital Universitario Clínico San Cecilio, E-18016 Granada, Spain; 4 Centre for Biomedical Research, University of Granada, E-18016 Granada, Spain; 5 Radiology and Physical Medicine Department, University of Granada, E-18016 Granada, Spain; ainhoa.perezcantero@gmail.com

**Keywords:** meconium, perfluoroalkyl substances, parabens, benzophenones, bisphenols, LC–MS/MS

## Abstract

Major concerns have been raised about human exposure to endocrine-disrupting chemicals (EDCs) during pregnancy. Effective methodologies for the assessment of this exposure are needed to support the implementation of preventive measures and the prediction of negative health effects. Meconium has proven a valuable non-invasive matrix for evaluating cumulative exposure to xenobiotics during the last two trimesters of pregnancy. The study objective was to develop a novel method to determine the presence in meconium of perfluoroalkyl substances (PFASs), bisphenols, parabens, and benzophenones, EDCs that are widely used in the manufacture of numerous consumer goods and personal care products, including cosmetics. Ten PFASs, two bisphenols, four parabens, and four benzophenones were measured in meconium samples prepared by using a combination of Captiva Enhanced Matrix Removal (EMR) lipid cartridges with salt-assisted liquid–liquid extraction (SALLE) and dispersive liquid–liquid microextraction (DLLME) before the application of liquid chromatography–tandem mass spectrometry (LC–MS/MS). Experimental parameters were optimized by applying different chemometric techniques. Limits of detection ranged from 0.05 to 0.1 ng g^−1^, and between-day variabilities (relative standard deviations) ranged from 6.5% to 14.5%. The method was validated by matrix-matched standard calibration followed by a recovery assay with spiked samples, obtaining percentage recoveries of 89.9% to 114.8%. The method was then employed to measure compounds not previously studied in this matrix in 20 meconium samples. The proposed analytical procedure yields information on cumulative in utero exposure to selected EDCs.

## 1. Introduction

Industrial activities since the latter part of the 20th century have resulted in the exposure of the entire biosphere, including humans, to a wide range of anthropogenic compounds. Human health can be negatively affected by exposure to some of these compounds, including endocrine-disrupting chemicals (EDCs). Over the past decade, hundreds of chemicals have been identified as EDCs, whose potential risks have been described in multiple in vivo and epidemiological studies, underscoring the need to elucidate and mitigate their impact on human health.

Pregnancy is one of the most vulnerable windows of exposure to EDCs, and some of these can alter normal embryonic and fetal development [1,2]. Thus, in utero exposure to EDCs has been associated with anomalous neonatal weight and head circumference values [3,4], urogenital malformations [5], reproductive development abnormalities [6,7], and cognitive and neurodevelopmental disorders [8,9]. Early exposure to EDCs has also been associated with health problems later in life, including male infertility [10], endometriosis [11], and even cancer and cancer progression [12,13,14].

Hence, there is a need for an effective method to assess EDC exposure during pregnancy for the development of early prevention measures and the prediction of potential health issues in the future. In this regard, meconium has emerged as a non-invasive matrix for evaluating cumulative prenatal exposure to EDCs. Meconium begins to accumulate in the fetal intestine at around week 12 of pregnancy and is excreted 24–72 h post-delivery; due to its low metabolic activity, it is considered to reflect exposure between months 4 and 9 of the pregnancy [15,16].

EDCs previously investigated in meconium samples include organophosphate pesticides, pyrethroids, carbamates [17,18,19], paraquat [20], polychlorinated biphenyl compounds (PCBs), polyaromatic hydrocarbons (PAHs) [21], phthalates [22,23], therapeutic drugs (e.g., ibuprofen, paracetamol, or atenolol), and caffeine [16]. However, there is little or no information available about the presence in meconium of many other substances with potentially harmful effects on health, leading to a lack of knowledge about fetal cumulative exposure to other xenobiotics with demonstrated or suspected disruptive activity. The present study focuses on four groups of EDCs whose presence in meconium has not previously been investigated: (1) perfluoroalkyl substances (PFAS); (2) bisphenol A (BPA) and bisphenol S (BPS); (3) parabens [methyl (MPB), ethyl (EPB), propyl (PPB), and butyl (BPB) paraben]; and (4) benzophenones [benzophenone 1 (BP-1), benzophenone 3 (BP-3), benzophenone-8 (BP-8), and 4-hydroxybenzophenone (4-OH-BP)]. PFASs are widely employed for furniture surface coatings, textiles, paper products, kitchenware, and food packaging materials, among others [24]. Bisphenols are the main monomers used to manufacture numerous products based on epoxy resins and/or polycarbonate plastics [25]. For their part, benzophenones are included in cosmetic products, personal care products (PCPs), sunscreens (as UV filter), and food packaging (to reduce light degradation) [26]. Finally, the low cost and broad-spectrum antimicrobial properties of parabens have led to their extensive application as preservatives in PCPs [27].

Meconium contains, in decreasing order of abundance, water (80%), lipids, proteins, intestinal epithelial cells, neonatal hairs (lanugo), and minerals [15]. This complexity poses an analytical challenge, especially in the context of human biomonitoring programs which require accurate and sensitive methods that can be implemented without the need for sophisticated equipment (e.g., ultrasound or accelerated solvent extraction devices). To date, EDCs in meconium samples have been investigated by using solid–liquid extraction (SLE), ultrasound-assisted extraction (UAE), and solvent pressurized liquid extraction (SPLE) followed by multiple solid-phase extraction (SPE) steps for the clean-up [16,28]. However, enzymatic liquefaction using keratinase is an appropriate strategy to deal with the sticky and pseudo-solid characteristics of meconium, given the substantive presence of lanugo and epithelial cells. Indeed, our team previously applied enzymatic liquefaction to determine selected EDCs in placental tissue, using collagenase as enzyme [29,30]. A liquefied matrix favors much closer contact with the extraction solvents and is eminently suitable for the application of salt-assisted liquid–liquid extraction (SALLE) and dispersive liquid–liquid microextraction (DLLME) techniques. A combination of these procedures has achieved high efficacy in the extraction of numerous chemical compounds from different complex matrices [31,32]. However, to our best knowledge, this approach has not yet been used to analyze EDCs in meconium. In the specific case of this biological compartment, which has a substantial lipid content, it may also be expedient to add a pre-cleaning step using specialized SPE devices such as Captiva Enhanced Matrix Removal (EMR) lipid cartridges. EMR-Lipid sorbent is designed to selectively interact with unbranched hydrocarbon chains of lipids, providing high analyte recovery and precision in fatty/lipid-rich matrices [33,34].

The objective of this study was to develop and apply a method to determine the presence in meconium of ten PFASs, two bisphenols, four parabens, and four benzophenones by liquid chromatography–tandem mass spectrometry (LC–MS/MS), using a combination of Captiva-EMR cartridges with SALLE and DLLME techniques to treat the samples. This procedure was implemented in 20 meconium samples obtained from anonymous donors. The results evidence the presence in meconium samples of PFASs and other phenolic EDCs (bisphenols, parabens, and benzophenones) that have not previously been investigated in this biological matrix.

## 2. Materials and Methods

### 2.1. Chemicals and Reagents

All reagents were of analytical grade unless otherwise specified. Bisphenols [bisphenol A (BPA), bisphenol S (BPS), and labeled deuterium bisphenol A (BPA-D_16_)], parabens [methylparaben (MPB), ethylparaben (EPB), propylparaben (PPB), butylparaben (BPB), methylparaben ring ^13^C_6_ labeled (MPB-^13^C_6_), ethylparaben ring ^13^C_6_ labeled (EPB-^13^C_6_), propylparaben ring ^13^C_6_ labeled (PPB-^13^C_6_), and butylparaben ring ^13^C_6_ labeled (EPB-^13^C_6_)], benzophenones [benzophenone-1(BP-1), benzophenone-3 (BP-3), benzophenone-8 (BP-8), 4-hydroxybenzophenone (4-OH-BP), labeled deuterium benzophenone-3 (BP-3-D_5_)], perfluorohexanoic acid (PFHxA), perfluoroheptanoic acid (PFHpA), perfluorooctanoic acid (PFOA), perfluorononanoic acid (PFNA), perfluorodecanoic acid (PFDA), perfluoroundecanoic acid (PFUnA), perfluorododecanoic acid (PFDoA), perfluorotridecanoic acid (PFTrA), perfluorohexane sulfonate (PFHxS), and perfluorooctane sulfonate (PFOS) were obtained from Sigma-Aldrich (Madrid, Spain). PFAS mass-labeled internal standards (^13^C_5_-PFHxA, ^13^C_4_-PFOA, ^13^C_2_-PFDA, ^13^C_2_-PFDoA, ^13^C_4_-PFOS) were supplied by Wellington Laboratories (Ontario, Canada). Water (18.2 MΩ cm) was purified using the Milli-Q system (Millipore, Bedford, MA, USA).

Individual solutions of target analytes and internal standards (1000 mg L^−1^) were prepared in acetonitrile and stored at 4 °C, proving to be stable for at least six months. Standard mixtures were prepared with acetonitrile. Sets of solutions ranging from 0.005 to 0.1 mg L^−1^ (PFASs) and from 0.02 to 1.0 mg L^−1^ (bisphenols, parabens, and benzophenones) were utilized for optimization, calibration, and validation purposes. Solutions of 0.01 mg L^−1^ in acetonitrile were prepared as mass-labeled internal standards for PFASs and solutions of 0.2 mg L^−1^ as mass-labeled internal standards for bisphenols, parabens, and benzophenones. Captiva EMR-Lipid cartridges (6 mL, 600 mg) were obtained from Agilent Technologies (Santa Clara, CA, USA), and HPLC-grade acetonitrile (ACN) and LC–MS grade ACN and trichloromethane (TCM) were purchased from Merck (Darmstadt, Germany). Phosphate buffer saline (PBS), sodium chloride, formic acid, ammonium acetate, and *Bacillus licheniformis* keratinase were supplied by Sigma-Aldrich (Madrid, Spain). The enzymatic solution was prepared by dissolving 0.1 mg of enzyme powder in 10 mL of PBS medium (0.01 M, pH = 7.4) immediately before its utilization.

### 2.2. Analytical Equipment and Software

LC–MS/MS analyses used a Shimadzu Nexera XR LC-20A liquid chromatography system (Shimadzu, Kyoto, Japan) coupled with an AB Sciex Triple Quadrupole (QqQ) MS/MS 5500 mass spectrometer (AB Sciex, Framingham, MA, USA), performing statistical analyses with Statgraphics Centurion XVI 16.0.07 (Manugistics Inc., Rockville, MD, USA). A Hei-MIX incubator 1000 (Heidolph Instruments GmbH & Co, Schwabach, Germany) was used for the keratinase enzymatic treatment.

### 2.3. Sample Collection and Storage

Meconium samples were collected from 20 newborns at the San Cecilio Hospital in Southern Spain (Granada). All parents signed their informed consent to the donation of meconium from their child. The study was approved by the hospital ethical committee. A wooden spatula was used by the attending nurse to gather the meconium sample directly from the first diaper of the newborn within 24 h of delivery, placing it in a 20 mL glass container for immediate storage at −80 °C.

### 2.4. Preparation of Positive Control (Spiked) Samples

The method was optimized and validated using a pool of five meconium samples from different children. Pooled samples were spiked at 0.4 ng g^−1^ (PFASs) and 4.0 ng g^−1^ (bisphenols, parabens, and benzophenones) for the optimization, and concentrations ranging from 0.1 to 2.0 ng g^−1^ (PFASs) and from 0.4 to 20.0 ng g^−1^ (bisphenols, parabens, and benzophenones) were used for the validation (calibration curves and recovery assays). Spiking was performed by pipetting 5 µL of the corresponding solution (see “Chemicals and Reagents” section) into 0.2 g aliquots of pooled sample. Mass-labeled internal standards were obtained by spiking samples at 0.5 ng g^−1^ for PFASs and 10 ng g^−1^ for phenolic compounds, using 10 µL of the corresponding solution.

### 2.5. Enzymatic Liquefaction of Samples

In this initial step, 0.2 g of meconium was placed in a glass centrifuge tube and 1.0 mL of the keratinase solution was added, followed by liquefaction at 50 °C for 4 h.

### 2.6. Sample Treatment

First, 8.0 mL acetonitrile was poured over the liquefied sample, and the resulting mixture was vortexed for 60 s and then centrifuged at 2600× *g* for 2 min. The supernatant was transferred to a Captiva EMR-Lipid cartridge previously conditioned with 6 mL water/acetonitrile (80:20, *v*/*v*). The eluates were collected by gravity in a polypropylene centrifuge tube for the SALLE-DLLME procedure. Accordingly, 320 mg NaCl and 35.5 µL of formic acid (98%) were added and the solution was manually shaken for 60 s; then, after centrifugation at 2600× *g* for 5 min, the supernatant was placed in a 15 mL screw-cap glass test tube, concentrated to 1 mL under a nitrogen stream, and diluted with 10.0 mL of 10% NaCl aqueous solution (*w*/*v*) at pH of 5. Next, 1.5 mL TCM was injected by syringe, and the mixture was shaken for 20 s and centrifuged for 5 min at 2600× *g*, transferring the whole sedimented phase into a glass vial for evaporation to dryness under a nitrogen stream. The residue was then dissolved with 80 µL of a 70:30 (*v*/*v*) mixture of water and acetonitrile, thereby preparing the sample for injection into the LC–MS/MS system.

### 2.7. Chromatography and Mass Spectrometry Conditions

Chromatography and mass spectrometry conditions were previously reported by our team [30]. 

A Gemini C18 column (100 mm × 2 mm i.d., 3 µm particle) from Phenomenex (Torrance, CA, USA) was used for chromatographic separation at an injection volume of 20 μL and column temperature of 25 °C. In the gradient mobile phase, 5 mM of an aqueous solution of ammonium acetate (pH = 6.5) served as solvent A and acetonitrile as solvent B. Gradient conditions were: 0.0–1.0 min, 30% B; 1.0–5.0 min, 30–60% B; 6.0–8.0 min, 70% B; 8.0–8.50 min, 70–90% B; and 8.50–9.50 min, 90% B; finally returning to 30% B in 0.1 min. The flow rate was set at 0.35 mL min^−1^, and the total duration of the run was 12.0 min.

Compounds were analyzed in negative ion mode using the selected reaction monitoring (SRM) mode with unit mass resolution for both Q1 and Q3. Mass spectrometry conditions were optimized by using solutions at a concentration of 50 µg L^−1^ for perfusion. The ion source temperature was set at 450 ºC and the capillary voltage at −4.5 kV. Nitrogen served as curtain gas at 35 psi and as ion source gas 1 and 2 at 40 psi. All electric potentials relevant to the spectrometric process were adjusted accordingly for each compound, and a dwell time of 20 ms was selected. Table 1 exhibits the optimal values for each compound and their respective diagnostic signals (SRM MS/MS transitions).

### 2.8. Quality Control

Background contamination was tested by using procedural blanks, which showed no quantifiable concentration of any target analyte. In addition, a pool of blank meconium samples was spiked with PFASs at 0.1 ng g^−1^, 0.2 ng g^−1^, 0.5 ng g^−1^, and 1.0 ng g^−1^ and with phenolic target compounds at 0.4 ng g^−1^, 1.0 ng g^−1^, 4.0 ng g^−1^, and 20.0 ng g^−1^, performing duplicate injections of spiked blank meconium samples every 15 injections.

## 3. Results and Discussion

### 3.1. Removal of Lipid Interferences by Captiva-EMR Cartridge

Aliquots of liquefied pool meconium aliquots were diluted with 8 mL ACN, a known solvent for this matrix removal strategy, because a ratio of 20:80 water/ACN is considered to provide optimal cartridge performance [33,34,35]. However, when eluates were concentrated under nitrogen stream and redissolved with the initial mobile phase, they yielded a turbid solution that was not suitable for injection into the LC–MS/MS system. The one-step sample treatment commonly associated with Captiva-EMR was therefore discarded, with the addition of a subsequent SALLE-DLLME procedure to improve matrix removal of the meconium samples. Conversely, application of a SALLE-DLLME procedure without a previous Captiva-EMR step also produced extracts that could not be injected into the LC–MS/MS system due to their pseudo-gel nature. Consequently, the Captiva-EMR/SALLE/DLLME triad was selected. 

### 3.2. Optimization of SALLE Conditions

**Figure 1:**
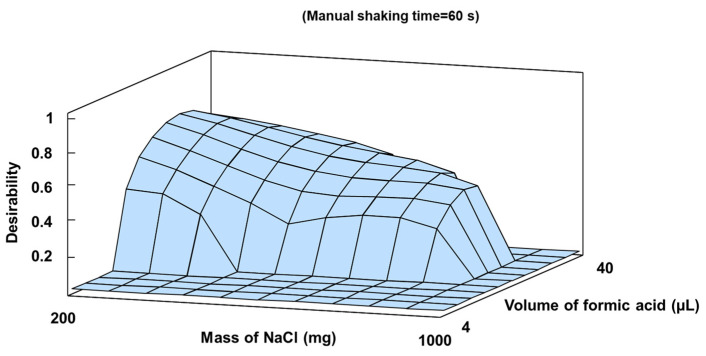
Estimated response surface for desirability function in SALLE optimization.

The mass of NaCl, the formic acid volume added, and the manual shaking time were optimized using a two-level factorial 2^3^ experimental structure with star points and six replicates at the central point, as summarized in Table S1. Maximum peak area values were obtained in different regions of the experimental domain for each analyte, applying the desirability function to obtain optimal values. This chemometric procedure was employed to determine optimal compromise values for experimental factors affecting multiple simultaneous responses [36]. A maximum desirability value of 0.72 out of an ideal value of 1 was achieved with 320 mg NaCl, 35.5 µL formic acid, and a 60 s shaking time. Figure 1 depicts the response surface associated with the desirability function obtained.

### 3.3. Optimization of DLLME Conditions

**Figure 2:**
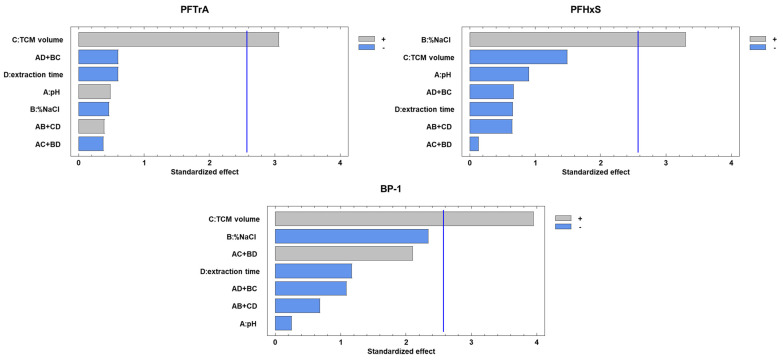
Pareto diagrams showing the significance of standardized effects of DLLME conditions on peak areas for PFTrA, PFHxS, and BP-1.

After establishing the SALLE conditions, multivariate experiments were conducted to optimize the response for each analyte in relation to the experimental DLLME parameters, i.e., pH of the aqueous solution, % NaCl in the aqueous solution, TCM volume, and extraction time. A two-level 2^4−1^ fractional factorial experimental design was used for this purpose, replicating the central point six times. The range and domain of this assay are detailed in Table S2. Notably, only the percentage of NaCl in the aqueous solution and the volume of TCM exhibited a positive influence on PFTrA, PFHxS, and BP-1 (Figure 2). *p*-values of the lack-of-fit tests were all >0.05, indicating the suitability of the experimental design to calculate the statistical significance of factors and their optimal value. Optimal values were found to be 10% NaCl in the aqueous solution and 1.5 mL TCM, setting the remaining experimental parameters at the most practical values, i.e., pH of 5 for the aqueous solution and extraction time of 20 s.

### 3.4. Analytical Performance and Method Validation

The linearity, sensitivity, accuracy (trueness and precision), and selectivity of the method were evaluated in accordance with US Food and Drug Administration guidelines [37].

The calibration function was established for each compound by testing ten concentrations, with four replicates for each, and plotting the analyte/mass-labeled surrogate peak area ratio against the corresponding analyte concentration, obtaining PFAS concentrations from 0.1 to 2.0 ng g^−1^ and phenolic compound concentrations from 0.4 to 20 ng g^−1^. The matrix effect (ME) was assessed by comparing the slopes of two calibration curves for each compound, generating one curve in milliQ water (W) and the other in meconium (M). The percentage ME was evaluated as follows:

ME (%) = [1 − (Slope of calibration in M/Slope of calibration in W)] × 100

ME values for all PFASs were negligible, ranging from −5.1% to 8.4%, whereas ME values for BPS, BP-1, BP-8, and 4-OH-BP were all above 30%. It was therefore necessary to conduct a matrix-matched calibration using a pool of five blank meconium samples, which were carefully selected from a larger set of donations that were not included as analyzed samples in the present study. According to validation guidelines, a sample is considered “blank” if its response for a target analyte is less than 20% of the response associated to the limit of quantification (LOQ). Figures S1 and S2 depict the chromatograms obtained from the blank meconium pool spiked at 0.5 ng g^−1^ for PFASs and 10.0 ng g^−1^ for bisphenols, parabens, and benzophenones.

#### 3.4.1. Accuracy (Precision and Trueness)

A recovery study involving spiked pooled meconium samples was conducted over three consecutive days. As depicted in Table 2, the precision of the method can be affirmed based on the relative standard deviation (RSD) values, all of which were below 15%. Similarly, the trueness of the method is substantiated by recovery values ranging from 89.9% to 114.8%.

#### 3.4.2. Limits of detection and quantification

The limit of quantification (LOQ) was defined as the lowest concentration at which trueness and precision were within ±20% and the limit of detection (LOD) as the lowest concentration at which signals were three-fold greater than background noise. Table 3 lists the LOQs and LODs for all studied EDCs.

#### 3.4.3. Linearity

A good linearity was observed, with *p*-values > 0.05 in the lack-of-fit test (P_lof_) and determination coefficients (R^2^) ranging from 99.1% to 99.6%. Consequently, a linear dynamic range (LDR) was established for concentrations ranging from the limit of quantification (LOQ) to 2.0 ng g^−1^ for PFASs and 20 ng g^−1^ for phenolic analytes (Table 3).

#### 3.4.4. Selectivity

The selectivity of the method was evaluated by analyzing the chromatograms of the procedure blank, detecting no interferences at the analyte retention times, as displayed in Figure S3. 

### 3.5. Method Application

The proposed method was employed to analyze the selected EDCs in 20 meconium samples. All analyzed samples exhibited detectable concentrations of various EDCs under study, as outlined in Table 4.

Out of the 11 PFASs investigated, only PFOA, PFDoA, and PFTrA were detected in the samples, with PFOA showing a high percentage of detection. Interestingly, PFOS was not detected in any sample, whereas its presence has been frequently described in the placenta [30,38,39]. This is the first investigation of PFAS levels in meconium, preventing comparisons with other reports.

BPA was detected in 50% of samples at concentrations ranging from undetected to 4.92 ng g^−1^, in line with the only previous study of BPA concentrations in meconium [40], in which BPA was detected in 46% of samples at a maximum concentration value of 3.93 ng g^−1^. In the present study, BPS was detected in only two samples (10%). 

Results for the selected parabens were similar to previous findings in the placenta [5], detecting MPB in 100% of meconium samples, followed by EPB (95)%, PPB (65%), and BPB (40%). However, they were markedly different to the report published by Cassoulet et al. [16], who observed an MPB detection rate of only 20% in 396 samples from a Canadian cohort. This discrepancy may be attributable to their higher LOD for this chemical (5 ng g^−1^ vs. 0.1 ng g^−1^). Cassoulet et al. [16] also described a much higher maximum MPB concentration (10,415 ng g^−1^) than in the present investigation (4.91 ng g^−1^), which may reflect differences in exposure patterns between the study populations. 

Finally, low detection frequencies were observed for BP-1 (25%) and BP-3 (30%), while neither BP-8 nor 4-OH-BP was detected in any sample. The presence of benzophenones in meconium has not previously been analyzed, preventing the comparison of results.

## 4. Conclusions

Concentrations of PFASs, bisphenols, parabens, and benzophenones were successfully measured in meconium samples from 20 newborns by preparing them with Captiva-EMR cartridges, SALLE, and DLLME before their analysis by LC–MS/MS. Experimental parameters were optimized by applying chemometric procedures and the procedure was duly validated. This study contributes evidence on the presence in meconium of PFASs, to our best knowledge for the first time, and of other phenolic EDCs that have been little studied in this matrix. The proposed procedure allows the determination of cumulative in utero exposure to EDCs and opens the way for research on its possible correlation with negative health effects.

## Figures and Tables

**Table 1:** SRM MS/MS transitions and optimized potentials.

Compound	Transitions	DP (V)	EP (V)	CE (V)	CXP (V)
PFHxA	313.0→269.0 ^a^ 313.0→119.0 ^b^	−43 −43	−8 −8	−12 −27	−10 −9
^13^C_5_-PFHxA	318.0→272.9 ^a^ 318.0→121.0 ^b^	−32 −32	−10 −10	−12 −30	−11 −10
PFHpA	363.0→319.1 ^a^ 363.0→169.0 ^b^	−40 −40	−8 −8	−12 −25	−9 −11
PFOA	413.0→369.0 ^a^ 413.0→219.2 ^b^	−40 −40	−9 −9	−13 −24	−10 −10
^13^C_4_-PFOA	417.1→371.8 ^a^ 417.1→222.1 ^b^	−44 −44	−10 −10	−12 −27	−9 −10
PFNA	463.0→419.0 ^a^ 463.0→219.2 ^b^	−40 −40	−10 −10	−13 −35	−10 −9
PFDA	513.1→469.0 ^a^ 513.1→269.2 ^b^	−51 −51	−9 −9	−13 −32	−10 −9
^13^C_2_-PFDA	515.0→470.1 ^a^ 515.0→220.2 ^b^	−46 −46	−9 −9	−15 −33	−9 −8
PFUnA	563.0→519.0 ^a^	−60	−9	−15	−10
	563.0→269.2 ^b^	−60	−9	−31	−10
PFDoA	613.0→569.0 ^a^	−62	−10	−15	−8
	613.0→319.1 ^b^	−62	−10	−36	−9
PFTrA	662.9→619.0 ^a^	−52	−11	−17	−9
	662.9→369.1 ^b^	−52	−11	−40	−8
^13^C_2_-PFDoA	615.0→570.0 ^a^	−54	−9	−14	−11
	615.0→320.2 ^b^	−54	−9	−32	−10
PFHxS	399.1→79.9 ^a^	−52	−11	−68	−13
	399.1→98.8 ^b^	−52	−11	−54	−10
PFOS	499.0→80.1 ^a^	−80	−10	−97	−9
	499.0→98.9 ^b^	−80	−10	−80	−11
^13^C_4_-PFOS	502.9→80.1 ^a^	−81	−10	−97	−11
	502.9→99.1 ^b^	−81	−10	−75	−10
BPA	227.1→212.0 ^a^ 227.1→133.0 ^b^	−80 −80	−10 −10	−26 −36	−5 −10
BPS	249.0→108.0 ^a^ 249.0→156.0 ^b^	−85 −85	−10 −10	−36 −30	−6 −10
BPA-D_16_	241.1→223.1 ^a^ 241.1→142.1 ^b^	−80 −80	−10 −10	−28 −36	−5 −10
MPB	150.9→136.0 ^a^ 150.9→107.9 ^b^	−64 −64	−10 −10	−20 −30	−5 −5
MPB−^13^C_6_	157.0→142.0 ^a^ 157.0→114.0 ^b^	−60 −60	−10 −10	−25 −30	−6 −5
EPB	164.9→136.0 ^a^ 164.9→107.9 ^b^	−62 −62	−10 −10	−20 −30	−6 −5
EPB-^13^C_6_	171.1→142.0 ^a^ 171.1→114.0 ^b^	-65 −65	−10 −10	−21 −35	−5 −5
PPB	179.1→136.0 ^a^ 179.1→107.9 ^b^	−68 −68	−10 −10	−20 −30	−5 −5
PPB-^13^C_6_	185.0→142.0 ^a^ 185.0→114.0 ^b^	−66 −66	−10 −10	−20 −28	−6 −6
BPB	193.1→136.1 ^a^ 193.1→107.9 ^b^	−75 −75	−10 −10	−20 −30	−5 −5
BPB-^13^C_6_	199.0→142.0 ^a^ 199.0→114.0 ^b^	−60 −60	−10 −10	−20 −30	−10 −6
BP-1	213.0→135.1 ^a^ 213.0→169.0 ^b^	−70 −70	−10 −10	−27 −27	−10 −6
BP-3	227.1→211.1 ^a^ 227.1→182.9 ^b^	−55 −55	−10 −10	−30 −50	−10 −10
BP-6	273.0→123.0 ^a^ 273.0→118.0 ^b^	−60 −60	−10 −10	−24 −50	−6 −10
BP-8	243.0→123.0 ^a^ 243.0→108.1 ^b^	−50 −50	−10 −10	−23 −50	−10 −7
4-OH-BP	197.1→119.1 ^a^ 197.1→168.8 ^b^	−60 −60	−10 −10	−32 −27	−10 −10
BP-3-D_5_	232.0→214.9 ^a^ 232.0→187.1 ^b^	−55 −55	−10 −10	−30 −50	−6 −10

^a^ SRM transition used for quantification; ^b^ SRM transition used for confirmation; DP: declustering potential; EP: entrance potential; CE: collision energy; CXP: collision cell exit potential.

**Table 2:** Recovery assay, precision, and trueness of the method.

	Spiked (ng g^−1^)	Found ^a^ (ng g^−1^)	Recovery (%)	RSD (%)		Spiked (ng g^−1^)	Found ^a^ (ng g^−1^)	Recovery (%)	RSD (%)
PFHxA	0.100	0.104	104.1	12.6	BPA	0.400	0.409	102.2	13.1
	0.200	0.225	112.5	13.8		1.000	1.097	109.7	10.7
	0.625	0.633	101.3	13.2		10.000	10.16	101.6	8.7
	1.00	1.102	110.2	12.9		20.000	21.15	105.7	10.1
PFHpA	0.100	0.103	102.9	14.1	BPS	0.400	0.406	101.5	14.5
	0.200	0.203	101.4	11.5		1.000	1.019	101.9	11.9
	0.625	0.698	111.6	12.1		10.000	10.51	105.1	7.6
	1.00	1.118	111.8	11.5		20.000	19.23	96.2	6.8
PFOA	0.100	0.106	105.7	12.8	MPB	0.400	0.419	104.8	11.4
	0.200	0.223	111.7	9.6		1.000	1.003	100.3	11.3
	0.625	0.679	108.6	13.9		10.00	11.060	110.6	10.2
	1.000	1.109	110.9	11.6		20.000	19.990	99.9	6.3
PFNA	0.100	0.109	109.1	12.4	EPB	0.400	0.428	107.1	11.8
	0.200	0.207	103.5	11.0		1.000	1.039	103.9	8.4
	0.625	0.611	97.8	8.4		10.000	11.180	111.8	9.2
	1.000	1.104	110.4	13.1		20.000	21.140	105.7	10.8
PFDA	0.100	0.097	97.2	12.1	PPB	0.400	0.418	104.7	12.9
	0.200	0.221	110.5	9.9		1.000	1.007	100.7	12.9
	0.625	0.601	96.1	8.1		10.000	11.278	112.8	4.8
	1.000	1.131	113.1	9.1		20.000	22.300	111.6	7.2
PFUnA	0.100	0.093	93.5	12.6	BPB	0.400	0.354	88.5	10.5
	0.200	0.180	89.9	13.3		1.000	0.975	97.5	8.3
	0.625	0.626	100.2	9.1		10.000	11.070	110.7	7.3
	1.000	0.972	97.2	7.7		20.000	22.150	110.7	5.3
PFDoA	0.100	0.115	114.8	14.3	BP-1	0.400	0.413	103.2	13.4
	0.200	0.226	113.0	13.9		1.000	1.095	109.5	11.8
	0.625	0.597	95.6	10.0		10.000	11.230	112.3	7.7
	1.000	0.918	91.8	11.6		20.000	19.840	99.2	5.9
PFTrA	0.100	0.103	103.1	13.3	BP-3	0.400	0.381	95.2	14.1
	0.200	0.200	100.0	13.5		1.000	1.045	104.5	9.8
	0.625	0.563	89.9	12.3		10.000	9.879	98.8	7.4
	1.000	0.937	93.7	11.6		20.000	21.231	106.2	8.1
PFHxS	0.100	0.109	109.2	14.2	BP-8	0.400	0.422	105.5	12.9
	0.200	0.223	111.5	13.1		1.000	1.108	110.8	11.4
	0.625	0.607	97.1	12.4		10.000	9.891	98.9	7.9
	1.000	1.078	107.8	9.1		20.000	21.120	105.6	6.8
PFOS	0.100	0.091	91.3	13.8	4-OH-BP	0.400	0.389	97.3	13.2
	0.200	0.198	99.1	9.7		1.000	1.091	109.1	10.6
	0.625	0.631	101.0	10.4		10.000	10.891	108.9	6.5
	1.000	0.972	97.2	8.7		20.000	21.432	107.2	7.1

^a^ Mean of 18 determinations.

**Table 3:** Analytical and statistical parameters.

	b (g ng^−1^)	s_b_ (g ng^−1^)	R^2^ (%)	LOD (ng g^−1^)	LOQ (ng g^−1^)	LDR (ng g^−1^)
PFHxA	1.692	0.023	99.3	0.05	0.10	0.10–2.0
PFHpA	1.935	0.030	99.2	0.05	0.10	0.10–2.0
PFOA	2.242	0.035	99.5	0.05	0.10	0.10–2.0
PFNA	1.773	0.021	99.3	0.05	0.10	0.10–2.0
PFDA	1.696	0.027	99.2	0.05	0.10	0.10–2.0
PFUnA	2.258	0.040	99.1	0.05	0.10	0.10–2.0
PFDoA	1.772	0.028	99.2	0.05	0.10	0.10–2.0
PDTrA	2.219	0.037	99.3	0.05	0.10	0.10–2.0
PFHxS	2.496	0.044	99.2	0.05	0.10	0.10–2.0
PFOS	1.623	0.021	99.2	0.05	0.10	0.10–2.0
BPA	0.243	0.004	99.4	0.10	0.40	0.40–20.0
BPS	0.077	0.002	99.6	0.10	0.40	0.40–20.0
MPB	0.157	0.002	99.6	0.10	0.40	0.40–20.0
EPB	0.139	0.001	99.5	0.10	0.40	0.40–20.0
PPB	0.135	0.001	99.6	0.10	0.40	0.40–20.0
BPB	0.150	0.001	99.4	0.10	0.40	0.40–20.0
BP-1	0.693	0.008	99.5	0.10	0.40	0.40–20.0
BP-3	0.175	0.003	99.3	0.10	0.40	0.40–20.0
BP-8	0.093	0.001	99.2	0.10	0.40	0.40–20.0
4-OH-BP	0.263	0.003	99.4	0.10	0.40	0.40–20.0

b, slope; s_b_, slope standard deviation; R^2^, determination coefficient; LOD, limit of detection; LOQ, limit of quantification; LDR, linear dynamic range.

**Table 4:** Application of the proposed method to meconium samples.

Sample	Found Concentration, ng g^−1^										
	PFOA	PFDoA	PFTrA	BPA	BPS	MPB	EPB	PPB	BPB	BP-1	BP-3
S01	0.14	ND	ND	2.80	ND	0.64	D	D	ND	ND	ND
S02	0.24	D	ND	0.55	ND	0.40	D	ND	ND	D	ND
S03	0.22	ND	D	1.55	ND	0.41	D	D	ND	ND	ND
S04	0.15	D	ND	ND	ND	1.23	0.42	1.04	D	ND	D
S05	0.21	ND	D	0.90	ND	0.42	D	0.49	ND	ND	D
S06	0.19	D	0.10	ND	ND	3.04	D	ND	ND	D	ND
S07	0.23	ND	D	2.65	D	0.52	D	D	D	ND	ND
S08	0.15	D	0.19	2.95	ND	D	D	ND	ND	ND	ND
S09	0.21	D	D	4.92	ND	4.91	5.50	D	D	ND	ND
S10	D	D	ND	ND	ND	D	D	D	ND	ND	ND
S11	0.20	ND	ND	ND	ND	0.48	D	0.65	0.40	ND	ND
S12	0.14	ND	ND	1.41	ND	0.42	D	1.23	0.41	ND	ND
S13	ND	ND	ND	4.66	ND	D	ND	D	ND	ND	ND
S14	0.24	ND	D	ND	ND	0.43	D	ND	D	ND	D
S15	ND	ND	D	0.65	ND	D	D	ND	ND	ND	ND
S16	ND	ND	ND	ND	ND	D	D	ND	ND	ND	ND
S17	D	ND	0.11	ND	ND	1.47	D	0.66	ND	D	ND
S18	D	ND	ND	ND	ND	D	D	D	D	0.44	0.45
S19	D	ND	D	ND	0.45	0.76	0.51	0.69	0.47	D	0.47
S20	0.11	ND	ND	2.14	ND	1.25	D	D	ND	ND	D
Det. (n, (%)) ^a^	17 (85)	6 (30)	10 (50)	10 (50)	ND	20 (100)	19 (95)	13 (65)	8 (40)	5 (25)	6 (30)
Median	0.14	ND	ND	0.60	ND	0.42	D	D	ND	ND	ND
C.range ^b^	ND-0.24	ND-D	ND-0.19	ND-4.92	ND-0.45	D-4.91	ND-5.50	ND-1.23	ND-0.47	ND-0.44	ND-0.47

ND, not detected (<LOD); D, detected (>LOD and <LOQ). ^a^ Detected; ^b^ Concentration range.

## Data Availability

Data are contained within the article and Supplementary Materials.

## Supplementary Materials

The following supporting information can be downloaded at: https://www.mdpi.com/article/10.3390/toxics12010075/s1, Figure S1: Chromatograms obtained from blank pool meconium spiked at 0.5 ng g^−1^ with PFAS; Figure S2: Chromatograms obtained from blank pool meconium spiked at 10.0 ng g^−1^ with bisphenols, parabens, and benzophenones; Figure S3: Procedural blank obtained from milliQ water. Table S1: Experimental domain and design matrix for SALLE optimization; Table S2: Experimental domain and design matrix for diagnosis of DLLME factors.
