# Supplementary material for: Multiclass Determination of Endocrine-Disrupting Chemicals in Meconium: First Evidence of Perfluoroalkyl Substances in This Biological Compartment
Source: Toxics. 2024 Jan 15;12(1):75. doi: 10.3390/toxics12010075 (PMC10819471; doi:10.3390/toxics12010075)
Supplement: Supplementary file 1 [file toxics-12-00075-s001.zip › Table S1.pdf]

Table S1. Experimental domain and design matrix for SALLE optimization.

Factor	Level	
	Low	High
Mass of NaCl (mg)	200	1000
Volume of formic acid (μL)	4	40
Manual shaking time (s)	20	60

Design matrix point	Mass of NaCl (mg)	Volume of formic acid (μL)	Manual shaking time (s)
1	320	12.5	28
2	600	12.5	28
3	320	31.5	28
4	600	31.5	28
5	320	12.5	52
6	600	12.5	52
7	320	31.5	52
8	600	31.5	52
9	200	22	40
10	1000	22	40
11	460	4	40
12	460	40	40
13	460	22	20
14	460	22	60
15	460	22	40
16	460	22	40
17	460	22	40
18	460	22	40
19	460	22	40
20	460	22	40
