# Supplementary material for: Multiclass Determination of Endocrine-Disrupting Chemicals in Meconium: First Evidence of Perfluoroalkyl Substances in This Biological Compartment
Source: Toxics. 2024 Jan 15;12(1):75. doi: 10.3390/toxics12010075 (PMC10819471; doi:10.3390/toxics12010075)
Supplement: Supplementary file 1 [file toxics-12-00075-s001.zip › Table S2.pdf]

Table S2. Experimental domain and design matrix for diagnosis of DLLME factors.

Factor	Level	
	Low	High
pH of aqueous solution	2	5
% NaCl of aqueous solution	2	10
Volume of TCM (mL)	0.5	1.5
Extraction time (s)	20	60

Design matrix point	pH of aqueous solution	% NaCl of aqueous solution	Volume of TCM (mL)	Extraction time (s)
1	2	2	0.5	20
2	5	2	0.5	60
3	2	10	0.5	60
4	5	10	0.5	20
5	2	2	1.5	60
6	5	2	1.5	20
7	2	10	1.5	20
8	5	10	1.5	60
9	3.5	6	1	40
10	3.5	6	1	40
11	3.5	6	1	40
12	3.5	6	1	40
13	3.5	6	1	40
14	3.5	6	1	40
