# Supplementary material for: Multiclass Determination of Endocrine-Disrupting Chemicals in Meconium: First Evidence of Perfluoroalkyl Substances in This Biological Compartment
Source: Toxics. 2024 Jan 15;12(1):75. doi: 10.3390/toxics12010075 (PMC10819471; doi:10.3390/toxics12010075)
Supplement: Supplementary file 1 [file toxics-12-00075-s001.zip › toxics-2808135-supplementary-figures.pdf]

Supplementary Materials

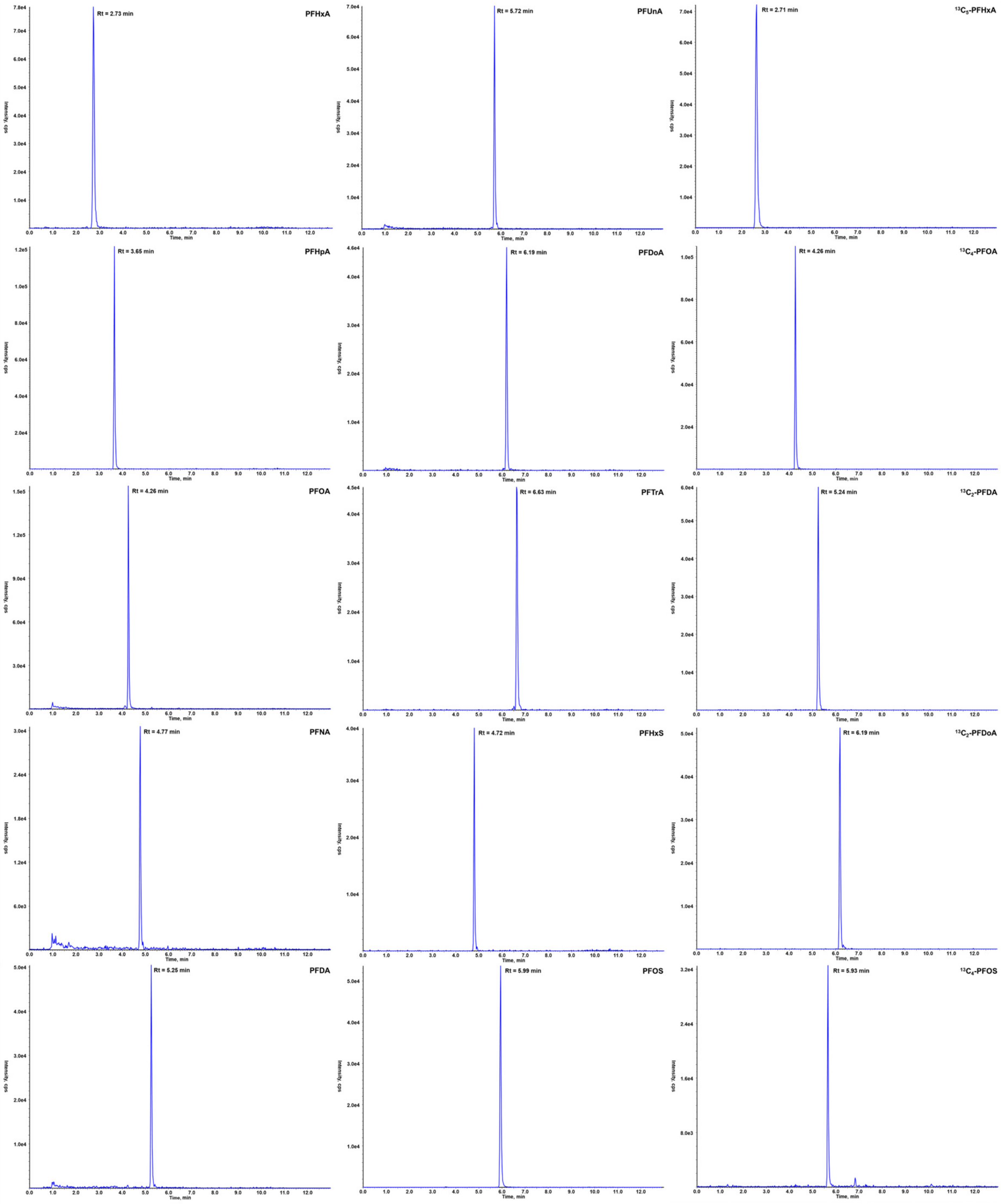

Figure S1. Chromatograms obtained from blank pool meconium spiked at 0.5 ng g^{-1} with PFAS.

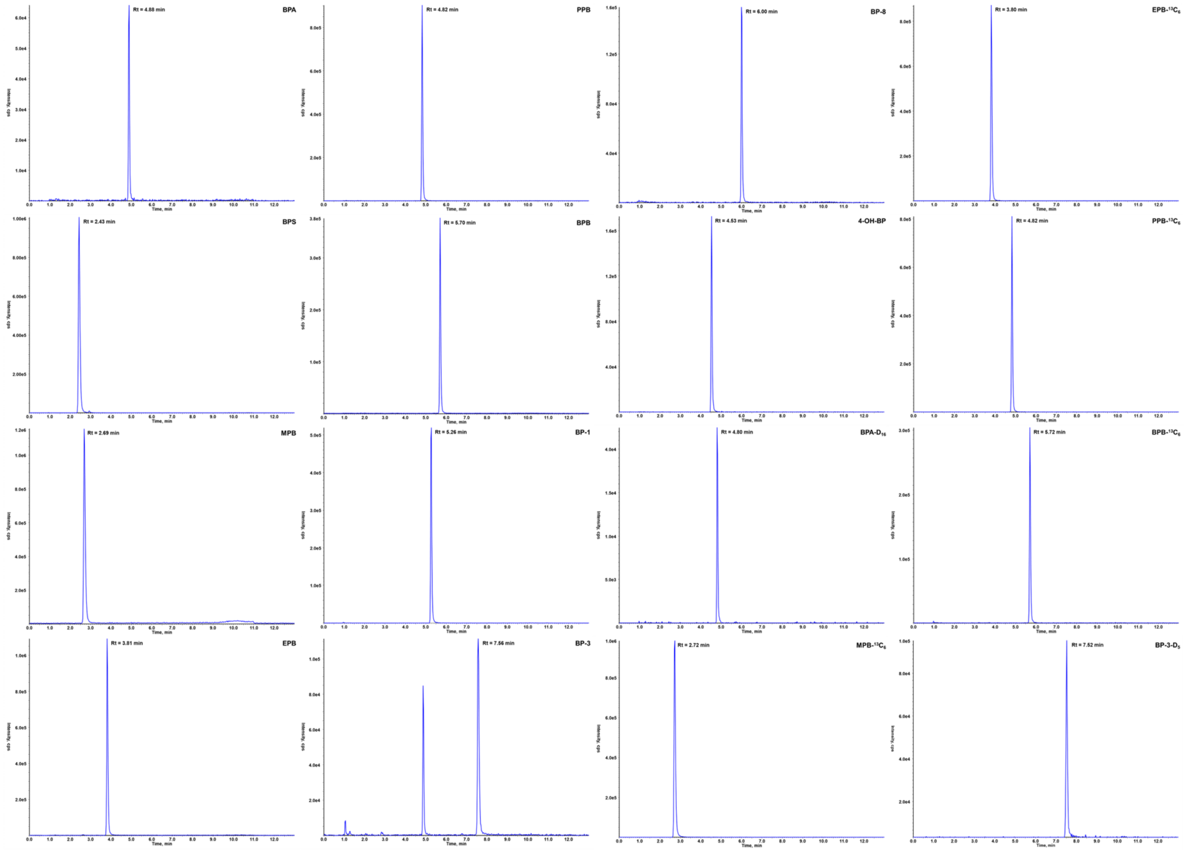

Figure S2. Chromatograms obtained from blank pool meconium spiked at 10.0 ng g^{-1} with bisphenols, parabens, and benzophenones.

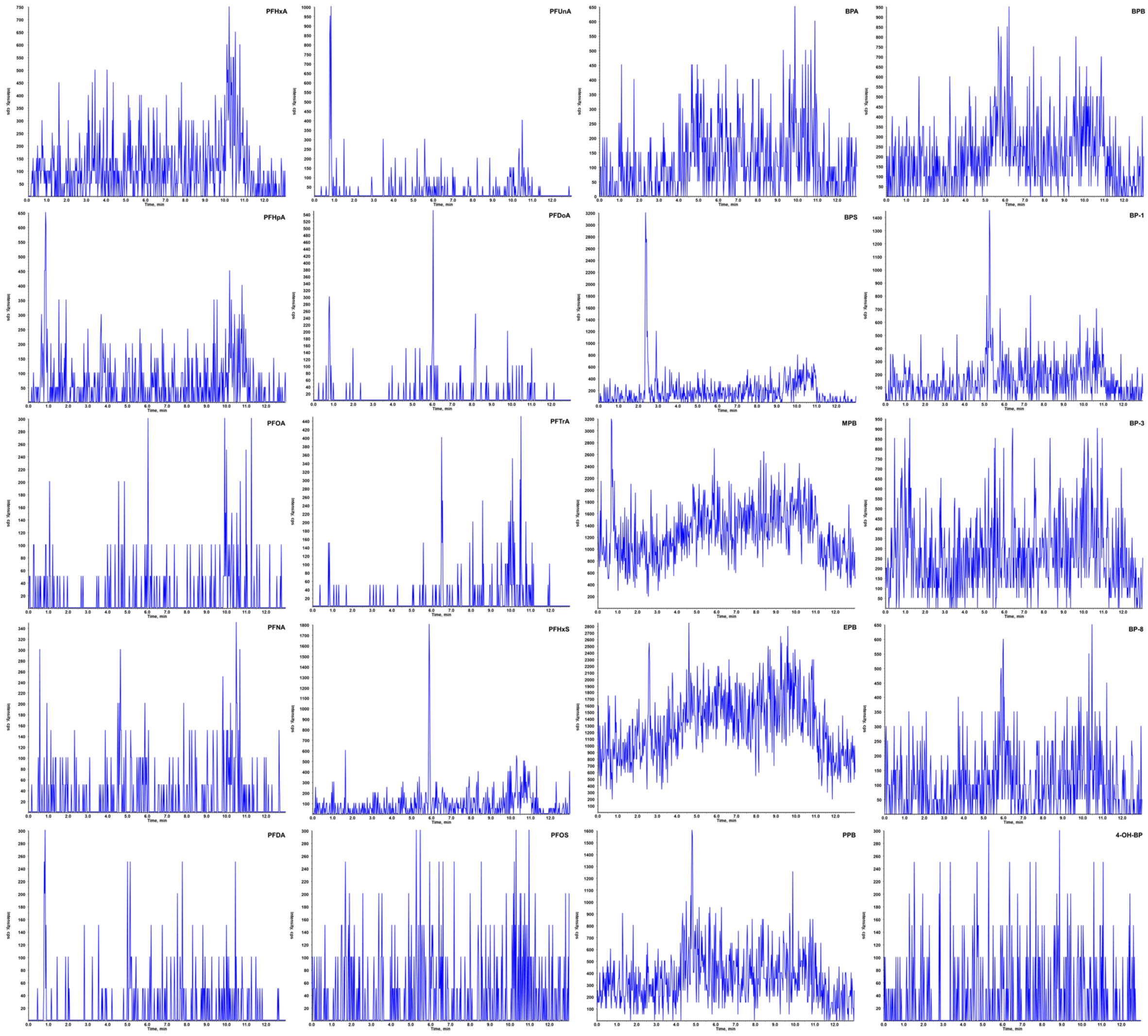

Figure S3. Procedural blank obtained from milliQ water.
